# Supplementary material for: Tracing Baculovirus AcMNPV Infection Using a Real-Time Method Based on ANCHORTM DNA Labeling Technology
Source: Viruses. 2020 Jan 2;12(1):50. doi: 10.3390/v12010050 (PMC7019957; doi:10.3390/v12010050)
Supplement: Supplementary file 1 [file viruses-12-00050-s001.zip › viruses-673599 supplementary/viruses-673599 supplementary.pdf]

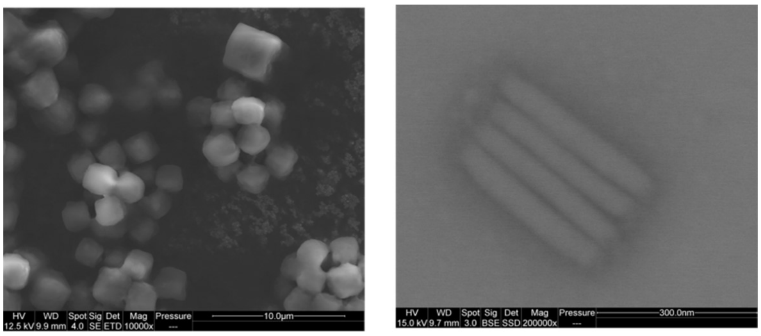

Figure S1. Electron microscopy observations of AcMNPV1-ANCHOR3 (a) Scanning electron microscopy of OBs and (b) Transmission electron microscopy of ODVs.
